# NURSING HOME TELEED INTERVENTION: ADVANCING NEW CARE MODELS

**DOI:** 10.1093/geroni/igz038.1225

**Published:** 2019-11-08

**Authors:** Charlene C Quinn, Anthony Roggio, Barr Erik, Ann Gruber-Baldini

**Affiliations:** 1 University of Maryland School of Medicine, Baltimore, Maryland, United States; 2 University of Maryland Medical Center, Baltimore, Maryland, United States; 3 University of Maryland Baltimore School of Medicine, Baltimore, Maryland, United States

## Abstract

New reimbursement and managed care models demonstrate the need to reduce avoidable Emergency Department (ED) use and limit preventable inpatient admissions for older adults in Skilled Nursing Facilities (SNF). The objective was to develop an ED telemedicine consultation intervention for SNF residents with acute medical problems. Secondary objectives including evaluation of health care utilization, provider satisfaction. Demonstration evaluation in three urban SNFs, telemedicine linked to university medical center ED. Mobile telemedicine cart equipment assessed SNF residents for any change in condition. ED physicians used tablets with secure access to conduct the resident assessment. Provider satisfaction measures imbedded in EMRs were completed at consultation visit end. 460 patients had changes in condition, 327 resulted in 911 calls, 85 deemed eligible for telemedicine consult. Conducted 57 telehealth consults. Forty (70%) telemedicine consult residents remained in the SNF. Fourteen residents were transferred to the ED. Average satisfaction scores were 5.8/7 for SNF nurses (n=49) and 5.6 for ED physicians (n=45). Lower-rated items related to technical equipment problems. ED physicians reported residents transferred to ED after telehealth visit had better continuity of care. The intervention was effective in preventing or delaying transfer of acutely ill, medically complex SNF residents. Implementation of the intervention identified need for SNF admission policy and procedure changes; weekly telemedicine training; SNF clinical advocates; on-site tracking and linkage of EMRs across providers; HIPAA shared medical record concerns. Future research plans include analyses of detailed SNF resident characteristics and business case assessment for reduction of transfers, ED and hospital utilization.

